# Effects of Low-Load Blood-Flow Restricted Resistance Training on Functional Capacity and Patient-Reported Outcome in a Young Male Suffering From Reactive Arthritis

**DOI:** 10.3389/fspor.2021.798902

**Published:** 2021-12-20

**Authors:** Stian Langgård Jørgensen, Inger Mechlenburg

**Affiliations:** ^1^Department of Occupational and Physical Therapy, Horsens Regional Hospital, Aarhus, Denmark; ^2^H-HIP, Horsens Regional Hospital, Horsens, Denmark; ^3^Department of Clinical Medicine, Aarhus University, Aarhus, Denmark; ^4^Department of Public Health, Aarhus University, Aarhus, Denmark

**Keywords:** venous occlusion, rehabilitation, exercise training, physical therapy, muscle venous occlusion, muscle

## Abstract

**Introduction:** Reactive arthritis (ReA) is a chronic inflammatory disease usually caused by a preceding gastrointestinal or genitourinary bacterial infection. ReA usually occurs in the lower limbs causing joint pain and joint swelling. Physiotherapy-led exercise is recommended to prevent muscle atrophy. The purpose of this case report is to describe the outcome after 12 weeks of low-load blood flow restricted resistance training (BFR-RT) as a rehabilitation method for a young male suffering from ReA.

**Methods and materials:** A 17-year-old male suffered from ReA in the both knee joints and the left hip joint. 36 months after the incident, he suffered from another ReA incident in his right knee. Non-steroid anti-inflammatory drugs and a new arthrocentesis added with corticosteroid injection was unsuccessful in treating the ReA. The patient performed 12 weeks of BFR-RT on the right lower limb with a low amount of supervision after the first week of training. Assessment of unilateral 30-sec chair stand test (u30-sec CST), low-thigh circumference above apex patella, The Knee Injury and Osteoarthritis Outcome Score (KOOS), The Forgotten Knee Joint Score (FJS), and Numeric Ranking Scale for pain (NRS) was performed at baseline and after 3,6,9, and 12 weeks of BFR-RT.

**Results:** The patient completed all planned exercise sessions. u30-sec CST improved with 7 repetitions (reps) on the right limb and 5 reps on the left leg. Low-thigh circumference decreased 1.1 cm on the right leg and 1.0 on the left leg. KOOS symptoms, ADL, quality of life and FJS demonstrated a clinically relevant change on 10, 14 and 23 points.

**Conclusion:** The present case study indicates that even with low amounts of supervision BFR-RT could increase functional performance, reduce knee joint swelling and improve key patient-reported outcome.

## Introduction

Reactive arthritis (ReA) is a chronic inflammatory disease usually occurring in the lower limb. ReA is often caused by a gastrointestinal or genitourinary bacterial infection which leads to a local immune reaction (Toivanen and Toivanen, 2004; Schmitt, 2017; Wendling et al., 2020). The incidence of ReA after infection varies with an incidence of 1-1.5% after digestive infection and 4-8% after genital Chlamydia infection (Wendling et al., 2020). Further, the duration of ReA-symptoms is normally six to 12 months. Unfortunately, up 30% of all patients suffering from ReA experience chronic arthritic symptoms, and as a part of the treatment, patients with ReA are recommended physiotherapy-led exercise to prevent skeletal muscle atrophy and joint stiffening (Wendling et al., 2020). However, due to the low incidence rate it is practically impossible to perform sufficiently powered randomized controlled trial to determine the most effective exercise modalities for this particular patient population. Therefore, information from smaller-scale studies, such as a case-report can add valuable information to the patient treatment.

Heavy resistance strength training (HRST) with loads corresponding to >75% of the one repetition maximum (1RM) is usually applied to promote skeletal muscle hypertrophy and skeletal muscle strength gains (Garber et al., 2011). HRST has consistently demonstrated to improve both skeletal muscle hypertrophy as well as muscle mechanical function in both healthy adults and patient populations across all age groups (Aagaard et al., 2002; Couppe et al., 2008; Suetta et al., 2008; Vissing et al., 2008; Skoffer et al., 2016; Calatayud et al., 2017; Ferraz et al., 2018). However, due to pain and joint swelling, HRTS may be contraindicated, rendering patients suffering from ReA to search for alternative exercise methods. During the last decade, research on resistance training with loads as low as 20% of 1RM with concurrent partial or complete blood flow restriction to the exercising limb (low-load blood-flow restricted resistance training: BFR-RT) has consistently proven to promote skeletal muscle hypertrophy and increase muscle strength comparable to that of HRTS (Wernbom et al., 2008; Hughes et al., 2017; Grønfeldt et al., 2020). The ability to promote muscle morphological and muscle mechanical adaptations with low loads makes BFR-RT very interesting in clinical rehabilitation (Hughes et al., 2017; Jørgensen et al., 2018, 2020; Petersson et al., 2020). Furthermore, BFR-RT is safe in both cardiac and orthopedic patient populations and leads to greater reduction in knee joint swelling (Hughes et al., 2017, 2019; Groennebaek et al., 2019; Patterson et al., 2019; Jørgensen et al., 2020). Results from our research group have demonstrated that patients can administer BFR exercises safely and correctly with minimal supervision (Petersson et al., 2020, Høghsholt et al., under review). Thus, BFR-RT may be feasible in patients with ReA to maintain or increase skeletal muscle mass and muscle mechanical function without exacerbating joint pain and/or joint swelling.

The purpose of this clinical case report is to describe the outcome after the use of BFR-RT as rehabilitation method for a young male suffering from ReA. We hope this case report will add to the existing literature on physiotherapist-led exercise methods aiming at maintaining and/or increasing functional capacity in patients suffering from ReA.

## Case Description

### History

**Figure 1:**
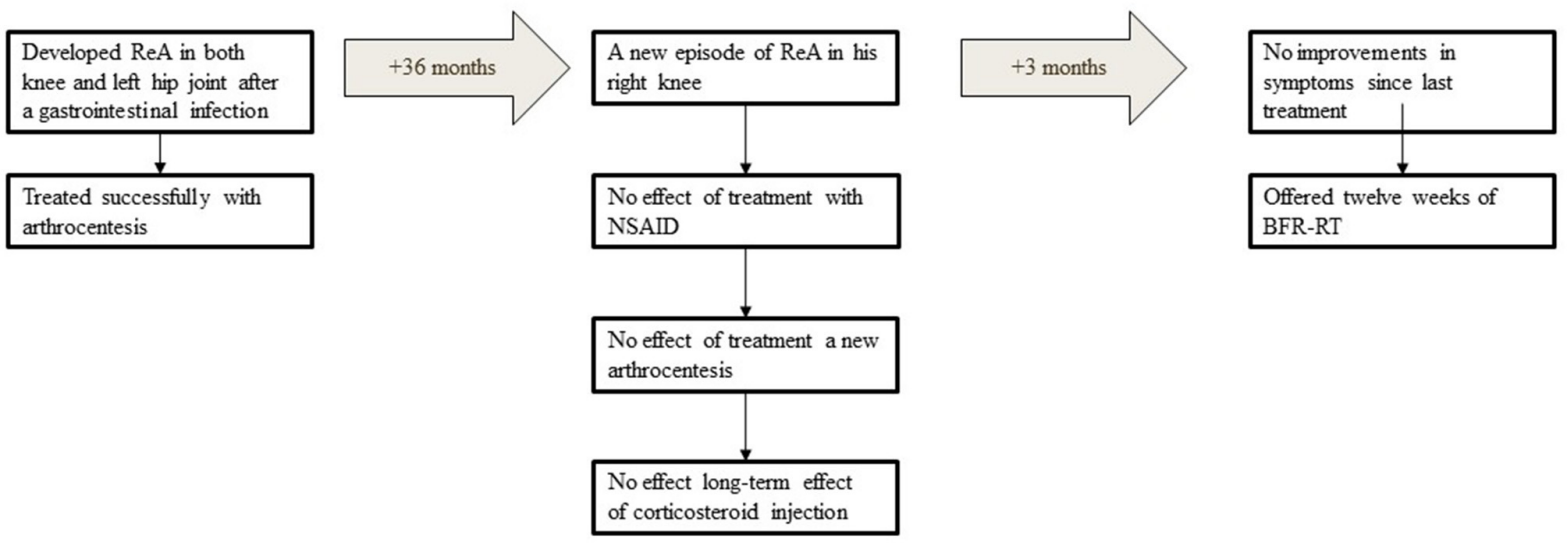
Timeline of the patient history. ReA, reactive arthritis; NSAID, non-steroid anti-flammatory drugs; BFR-RT, blood flow restricted exercise.

The patient was a formerly healthy 17-year-old young male (weight: 74.5 kg; height: 185 cm. brachial systolic/diastolic blood pressure: 129/78) with no family history of inflammatory joint- or connective tissue diseases. Further, the patient did not suffer from any cardiovascular diseases. After a week of sickness due to a gastrointestinal infection the patient developed ReA in both of his knee joints as well as the left hip joint, hence resulting in hip- and knee joint pain as well as joint swelling, ultimately reducing his physical activity level and quality of life. The ReA of hip joint as well as the knee joints was successfully treated with arthrocentesis, with full recovery in knee joint mobility and only small reduction in hip flexion and hip internal rotation as compared with the right hip joint. At the physical examination at the hospital, the range of motion was not quantified, but only compared between each limb. Thirty-six months after the first ReA, the patient suffered a relapse in his right knee. Similar to the episode, he suffered from knee joint pain and knee joint swelling which resulted in a reduced physical activity-level as well as a reduced quality of life. Treatment with non-steroid anti-inflammatory drugs (NSAID) did not reduce symptoms and a new arthrocentesis and aorticosteroid injection only reduced the knee swelling temporary. After 3 months without improvements, the patient was offered 12 weeks of BFR-RT to (i) improve his functional capacity, (ii) reduce swelling of the knee joint, and (iii) reduce knee symptoms (Figure 1). The patient accepted to engage in the study and signed a written informed consent in accordance with the Helsinki Declaration. According to Danish law, case studies do not require formal ethical approval.

### Intervention

**Figure 2:**
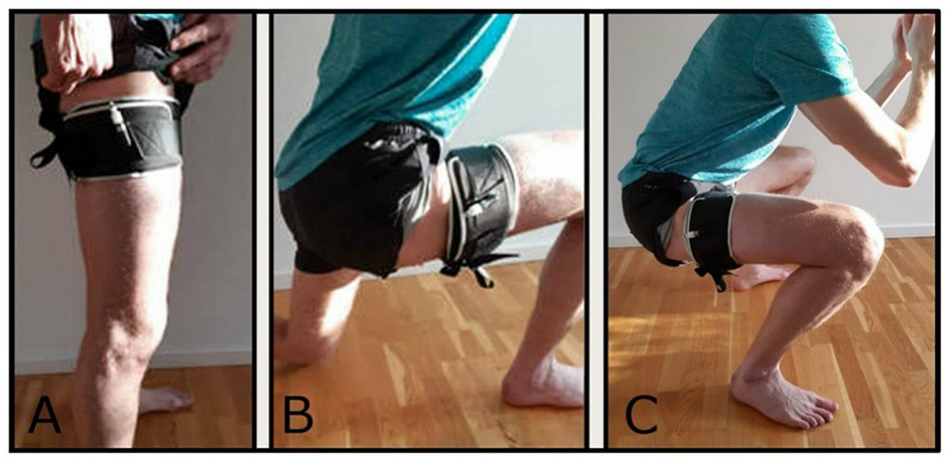
**(A)** Placement of the pneumatic cuff; **(B)** illustration of the lunge exercise; **(C)** illustration of the squat exercise.

The BFR-RT was performed at home every second day for 12 weeks, and consisted of squat and lunges (Figures 2B,C). The load in each exercise was the body weight. A conically shaped pneumatic cuff (Occlude APS, size: Large, width = 11.7 cm) was placed around the proximal part of the right thigh (Figure 2A). Each exercise was performed in 4 sets with 30 repetitions (reps) in round one and 15 reps in round two, three, and four. Each set was interspaced with 30 sec rest and each exercise was separated by a 5-min rest pause. A physiotherapist supervised the exercises during the first week of exercise (Table 1). The patient was instructed to apply the cuff correctly, to inflate/deflate the cuff, and check that the inflation was kept constant during the entire duration of each exercise. Also, the patient was carefully instructed in how to perform the exercises correctly. The cuff pressure was an absolute pressure and not determined based on the total limb occlusion pressure. As we decided to maintain a preset repetition scheme (30-15-15-15) and use body weight as load, we decided to gradually increase the cuff pressure with 10 mmHg from week 1 to week 6 (110–150 mmHg). For safety reasons the cuff pressure was kept constant from week 6 to week 12. Immediately after the last set of each exercise, the cuff was deflated.

**Table 1:** Exercise variables.

**Exercise variable**	
Blood flow restriction	110–150 mmHg
Cuff width	11.7 cm
Sets	4
Load intensity	Body weight
Repetitions 1st set	30
Repetitions 2nd, 3rd, and 4th set	15
Contraction modes per repetition	
Range of motion	Maximum
Rest between sets	30 sec
Rest between exercises	5 mins
Rest between sessions	24 h

### Outcome Measures

One physiotherapist collected outcome measures at baseline and after 3, 6, 9, and 12 weeks of BFR-RT and included unilateral 30-Sec chair-stand test (u30-sec CST) (Thongchoomsin et al., 2020; Waldhelm et al., 2020) and low-thigh circumference with tape measure (Jakobsen et al., 2010) proximal to the upper edge of the patella in standing position. Also, the patient completed the Knee Injury and Osteoarthritis Outcome Score (KOOS) (Roos and Lohmander, 2003) and the Forgotten Knee Joint Score (FJS) (Behrend et al., 2012).

*The u30-sec CST* is a functional test used to assess lower limb strength (Waldhelm et al., 2020). Before testing, the physiotherapist demonstrated the movement after which the participant performed two practice repetitions to demonstrate the understanding of the test. From sitting on a 45 cm high chair, the patient performed as many single-leg sit to stands with full hip- and knee extension as possible in 30 Sec with the arms crossed in front of the chest. The patient descended until the buttock made contact with the chair. The patient was allowed to reverse the downward-movement as soon as he felt the chair. Thus, he did not have to bear weight through his buttock. Only repetitions correctly performed repetitions (i.e., as described above) were counted. (Waldhelm et al., 2020).

Low-thigh circumference was measured with the patient laying prone on an examination table. To measure low-thigh circumference, a tape measure was placed around the proximal border of the patella while the patient lay relaxed without contraction his knee extensor muscles. A reduction in circumference from baseline to follow-up would represent less joint swelling. Assessing low-thigh circumference with a tape measure has previously been demonstrated to be reliable and reproducible in patients suffering from osteoarthritis (Silva et al., 2014).

*The KOOS-questionnaire* was completed by the patient prior to the functional tests on each testing day. The patient completed the questionnaire in quiet and undisturbed environment with the possibility to ask the physiotherapist in charge of testing whenever he had questions. The KOOS is a patient-administered knee specific questionnaire comprising five subscales: Pain; Symptoms; Activities of daily living; Sport & Recreation; and Knee-Related Quality of Life. All questions are related to the patients' experiences the last seven days. Each item is scored from 0 to 4 (Roos and Lohmander, 2003). The raw score for each of the five subscales is the total sum of the associated item scores. Scores are transformed to a 0 to 100 scale. The scores of the five subscales are expressed as a composite outcome profile, higher scores indicate fewer problems, and a 10-point change in a subscale score is considered to represent a clinically meaningful change (Nilsdotter et al., 2003; Lyman et al., 2018).

*The FJS* consists of 12 questions with a five-point Likert response format from 0-4 point (Behrend et al., 2012). The FJS was completed prior to functional performance tests and after completing the KOOS questionnaire on each testing day. As with the KOOS questionnaire, the patient completed the questionnaire in an undisturbed and quiet environment with the possibility to ask the physiotherapist in charge of testing whenever he had questions. The score is transformed into a 0 to 100-point scale with high scores indicating good outcomes (i.e., being less aware of the knee during every day activities) (Behrend et al., 2012, 2017).

*The numeric rating scale for pain (NRS)* was used to quantify the level of pain prior to each testing session (Hawker et al., 2011). Thus, prior to completing the KOOS questionnaire, the patient reported the level of pain experienced in each knee while sitting relaxed in a chair with ~90 degree knee flexion on a scale from 0 to 10, where 0 represented no pain at all, and 10 represented the worst imaginable pain (Hawker et al., 2011).

## Data Analysis

Differences from pre- to post-intervention in repetitions, low-thigh circumference, KOOS- and FJS-scores and NRS scores were determined as both absolute change (*post score*−*baseline score* = *absolute change*) and relative change in percent ($\frac{post\;score-baseline\;score}{post\;score}\times 100$). Adherence was calculated as $\frac{\frac{Sessions\;completed}{week\;completed}}{session\;scheduled}\times 100=Adherence\;\left(\%\right)$. The statistical analysis was conducted in Stata 17.0 (StataCorp, TX, USA).

The manuscript was written in accordance with the [(Riley et al., 2017)CARE] guidelines.

## Results

The participant completed all planned exercise sessions (100% adherence) and all planned outcome assessments sessions (100% adherence).

Pre-to-post improvements in u30-sec CST was demonstrated for both the right and the left lower limb. However, a two-fold relative improvement in lower limb function was observed for the right lower limb (Table 2). Low-thigh circumference decreased equally on both the right and left thigh (Table 2), while a pain-level corresponding to 0 NRS was maintained throughout the intervention period (Table 2).

**Table 2:** Outcome measure from baseline to after 12 weeks of BFR-RT.

**Outcome measure**			**Baseline**	**3 weeks**	**6 weeks**	**9 weeks**	**12 weeks**	**Absolute** **difference**	**% Change**
u30-sec CST	Right	reps	10	14	14	14	17	7	41%
u30-sec CST	Left	reps	13	15	15	17	18	4	28%
Low-thigh circumference	Right	cm	41	40.5	40.4	40.4	39.9	1.1	−3%
Low-thigh circumference	Left	cm	38.4	38	37.6	37.8	37.4	1.0	−3%
Knee pain	Right	NRS	0	0	0	0	0		0%
Knee pain	Left	NRS	0	0	0	0	0		0%
KOOS									
Pain		Points	94	94	94	92	94	0	0%
Symptoms		Points	54	54	57	68	64	10	16%
ADL		Points	82	82	93	94	96	14	15%
Sport and recreation		Points	60	70	70	65	65	5	8%
Quality of life		Points	56	69	69	69	69	13	19%
FJS		Points	21	35	44	40	44	23	15%

*u30-sec CST, unilateral 30-sec chair stand test; KOOS, Knee Osteoarthritis Outcome Score; FKJS, Forgotten Knee Joint Score; reps, repetitions; cm, centimeters; NRS, Numeric Ranking Scale for Pain*.

KOOS subscales Symptoms, Activities of daily living (ADL), and Quality of Life (QOL) displayed a ≥10-point change from baseline to after 12 weeks of BFR-RT. Furthermore, a 23-point reduction in the FJS was demonstrated (Table 2).

## Discussion

After 12 weeks of BFR-RT every second day, the young male patient suffering from ReA reported and also demonstrated increased functional capacity, a reduction of knee symptoms, increased ability to perform ADL-activities, an improved QOL in line with reducing his awareness of the knee joint after 12 weeks of BFR-RT. Also, 12 weeks of BFR-RT did not provoke additional knee pain or increase knee joint swelling during the exercise period. Therefore, BFR-RT performed as home-based, body weight exercises seems both feasible, safe, and clinically relevant for patients suffering from ReA.

To our best knowledge, this is the first study to demonstrate that 12 weeks of bodyweight BFR-RT every second day was safe and feasible as an exercise method for increasing function and reduce knee joint swelling in a patient suffering from ReA. The reduction in knee swelling was lower than the findings in a RCT performed by Hughes et al. (2019) who reported a 5.8% reduction in knee joint swelling after 12 weeks of BFR-RT in patients with anterior cruciate ligament-reconstruction. However, some of the difference might be due to differences in measuring point as we measured just above the proximal border of the patellar, while Hughes and co-workers measured knee joint swelling at mid-patella level (Hughes et al., 2019). As a similar reduction in knee joint swelling was reported in both knee joints, it seems plausible that the low load applied during exercise (body weight only) was the primary reason for the reduction in knee joint swelling. However, we cannot rule out that the pre-to-post difference is due to test-retest variability. Test-retest variability difference in mid-thigh-circumference has been reported to be −0.3 ± 0.5 cm (Jakobsen et al., 2010), while Hughes et al. (2019) reported a standard error of mean (SEM) was 0.04 cm.

After the intervention period, the patient had improved lower limb function (improved u30-sec CST), suggesting that the exercise method increased lower limb strength and muscular power (Alcazar et al., 2020; Waldhelm et al., 2020). The increased functional performance, measured as an improved unilateral sit-to-stand function, may be due to increased lower limb strength. As such exercising with BFR has been suggested to cause tissue hypoxia, an increment in metabolites, and muscle cell swelling, which all contributes to increased protein synthesis, increased type II muscle fiber recruitment, local and systemic anabolic hormone synthesis, and stimulation of myogenic stem cells (Wernbom et al., 2008; Nielsen et al., 2012; Wernbom and Aagaard, 2019; Vopat et al., 2020). Thus, the gains in muscle strength as a result of exercising, would most likely translate into an improved functional performance. This contrasts the findings in Jakobsgaard et al. (2018) who were unable to find any change in muscle strength in 6 young males after 6 weeks of BFR-RT, although they demonstrated significant improvements in skeletal muscle hypertrophy of the vastus lateralis muscle. However, Yokokawa et al. (2008) demonstrated in a randomized trial increased isometric quadriceps strength and physical function after 8 weeks of BFR-RT with body weight as resistance compared to dynamic balance training in healthy elderly people (Yokokawa et al., 2008). Thus, BFR-RT with body weight can plausibly be considered a valid exercise alternative for increasing skeletal muscle strength in cases where HRST are contraindicated or impossible due to external circumstances (i.e., COVID-19 social restriction).

Importantly, despite exercising every second day for 12 weeks, these improvements were attained without inducing knee pain or increasing knee joint swelling indicating that present BFR-RT protocol was tolerable without overloading the knee joint and the surrounding structures.

Three of 5 KOOS subscales improved with at least 10 point and FJS improved 23 point after 12 weeks of BFR-RT also indicating that the patient benefitted from the BFR-RT protocol. This is in line with other studies utilizing BFR as an exercise treatment (Tennent et al., 2017; Ferraz et al., 2018). Ferraz et al. conducted a three-armed RCT presented demonstrated an pre-to-post improvement in all WOMAC subscales after 12 weeks of BFR-RT in patients suffering from knee OA (Ferraz et al., 2018). Also, Tennent et al. (2017) performed a pilot RCT and found significant improvements in all KOOS subscales after 12 sessions of postoperative BFR-RT in younger patients recovering from non-reconstructive arthroscopy (Tennent et al., 2017). Thus, based on the findings in our case study as well as findings from the above mentioned studies, it seems plausible that BFR-RT can induce functional improvements and increase patient-reported outcomes.

### Limitations

Some limitation to the present study needs to be addressed. The inherent limitations of a case report with only one participant renders any firm conclusions on the efficacy of the exercise method. However, due to the low prevalence of ReA it can be difficult to include several participants. Therefore, we consider the present case report important to both (i) demonstrate that BFR-RT was feasible as home-based exercise rehabilitation and (ii) improved functional performance and patient-reported outcomes. Furthermore, the exercise protocol utilized in the present study withholds some limitations that needs to be addressed. First, we decided to include a bilateral exercise (squat) while only restriction blood flow to the right lower limb. As the BFR accelerates the fatigue during an exercise compared to performing the same exercise without BFR (Counts et al., 2016; Loenneke et al., 2017; Jessee et al., 2018; Mattocks et al., 2018), the free-flow limb may compensate for the BFR-limb as it fatigues during the exercise. Therefore, we do know the extent the BFR-limb reached a true fatiguing state. To our best knowledge, the application of unilateral BFR during a bilateral exercise has only been performed in a few studies (Kilgas et al., 2019; Høghsholt et al., under review), both of which demonstrated improved functional performance after 8 weeks of exercise. Secondly, we used an absolute pressure to restrict blood flow to the exercising limb which gradually increased from 110 mmHg to 150 mmHg during the first 6 weeks of exercise. As we decided to utilize a preset repetition scheme while not adding any external load during the exercise period, we decided to restrict the blood flow gradually to increase the intensity of the exercise. This is in line with a previous study by Jakobsgaard et al. (2018) who utilized body weight sit to stand with BFR in healthy young males, increased the blood flow restriction during the intervention period (from 100 to 150–180 mmHg) to decrease the number of repetitions performed in each session. Additionally, Dankel et al. (2017), found that higher BFR pressures increased fatigue verified as decrements elbow flexor torque at very low loads (10–20% 1RM) without any differences in total training volume (load × repetitions). Thus, based on both Dankel et al. (2017) and Jakobsgaard et al. (2018) we wanted to utilize BFR pressure to increase the exercise intensity rather than adding external load to the exercises. Thirdly, as we used an absolute BFR pressure, we do not know if the applied pressure is within the recommended recently recommended range of 40–80% of total limb occlusion pressure (Patterson et al., 2019). To increase safety of prescribing BFR-RT as a home-based exercise method, we would recommend future studies to apply a relative pressure. However, similar absolute pressures have previously been applied in healthy young people with reporting any adverse events (Nielsen et al., 2012; Jakobsgaard et al., 2018). Therefore, we considered the present exercise protocol as safe to perform as a home-based rehabilitation program.

### Clinical Application

The exercise protocol applied in the present study demonstrated to be feasible and safe in this particular patient. Furthermore, the patient was able to perform the protocol at home without daily supervision, hence rendering the necessity for frequent inpatient visits. Thus, with relatively few supervised sessions, BFR-RT can be considered a clinically relevant exercise method for patients in need of rehabilitation to increase muscle function.

In conclusion, the present study indicates that BFR-RT can be performed safely with high adherence in patients suffering from ReA to increase functional performance, reduce knee joint swelling, and improve patient-reported outcomes. Future studies are required to compare the present exercise protocol performed with and without BFR to determine the necessity of BFR during these body weight exercises.

## Data Availability Statement

The original contributions presented in the study are included in the article/supplementary material, further inquiries can be directed to the corresponding author/s.

## Ethics Statement

Ethical review and approval was not required for the study on human participants in accordance with the local legislation and institutional requirements. The patients/participants provided their written informed consent to participate in this study. Written informed consent was obtained from the individual(s) for the publication of any potentially identifiable images or data included in this article.

## Author Contributions

IM: contributed to conception, design of the study, organized the database, and wrote sections of the manuscript. SJ: performed the statistical analysis and wrote the first draft of the manuscript. All authors contributed to manuscript revision, read, and approved the submitted version.

## Conflict of Interest

The authors declare that the research was conducted in the absence of any commercial or financial relationships that could be construed as a potential conflict of interest.

## Publisher's Note

All claims expressed in this article are solely those of the authors and do not necessarily represent those of their affiliated organizations, or those of the publisher, the editors and the reviewers. Any product that may be evaluated in this article, or claim that may be made by its manufacturer, is not guaranteed or endorsed by the publisher.
